# Supplementary material for: A fault-intrusion-tolerant system and deadline-aware algorithm for scheduling scientific workflow in the cloud
Source: PeerJ Comput Sci. 2021 Nov 2;7:e747. doi: 10.7717/peerj-cs.747 (PMC8576554; doi:10.7717/peerj-cs.747)
Supplement: Supplemental Information 6 [file peerj-cs-07-747-s006.zip › Myproject1.1/docs/org/cloudbus/cloudsim/class-use/Vm.html]

Uses of Class org.cloudbus.cloudsim.Vm


JavaScript is disabled on your browser.


- Overview
- Package
- Class
- Use
- Tree
- Deprecated
- Index
- Help

- Prev
- Next

- Frames
- No Frames

- All Classes

## Uses of Class org.cloudbus.cloudsim.Vm

- Packages that use Vm

  | Package | Description |
  | --- | --- |
  |  |  |
  | --- | --- |
  | org.cloudbus.cloudsim |  |
  | org.cloudbus.cloudsim.examples |  |
  | org.cloudbus.cloudsim.examples.power |  |
  | org.cloudbus.cloudsim.lists |  |
  | org.cloudbus.cloudsim.network.datacenter |  |
  | org.cloudbus.cloudsim.power |  |
  | org.cloudbus.cloudsim.power.lists |  |
  | org.cloudbus.cloudsim.provisioners |  |
  | org.workflowsim |  |
- - ### Uses of Vm in org.cloudbus.cloudsim

    Fields in org.cloudbus.cloudsim with type parameters of type Vm

    | Modifier and Type | Field and Description |
    | --- | --- |
    |  |  |
    | --- | --- |
    | `protected java.util.List<? extends Vm>` | DatacenterBroker.`vmList` The vm list. |
    | `protected java.util.List<? extends Vm>` | DatacenterBroker.`vmsCreatedList` The vms created list. |

    Methods in org.cloudbus.cloudsim with type parameters of type Vm

    | Modifier and Type | Method and Description |
    | --- | --- |
    |  |  |
    | --- | --- |
    | `<T extends Vm>  java.util.List<T>` | Datacenter.`getVmList()` Gets the vm list. |
    | `<T extends Vm>  java.util.List<T>` | DatacenterBroker.`getVmList()` Gets the vm list. |
    | `<T extends Vm>  java.util.List<T>` | Host.`getVmList()` Gets the vm list. |
    | `<T extends Vm>  java.util.List<T>` | DatacenterBroker.`getVmsCreatedList()` Gets the vm list. |
    | `protected <T extends Vm>  void` | Datacenter.`setVmList(java.util.List<T> vmList)` Sets the vm list. |
    | `protected <T extends Vm>  void` | DatacenterBroker.`setVmList(java.util.List<T> vmList)` Sets the vm list. |
    | `protected <T extends Vm>  void` | DatacenterBroker.`setVmsCreatedList(java.util.List<T> vmsCreatedList)` Sets the vm list. |

    Methods in org.cloudbus.cloudsim that return Vm

    | Modifier and Type | Method and Description |
    | --- | --- |
    |  |  |
    | --- | --- |
    | `Vm` | Host.`getVm(int vmId, int userId)` Returns a VM object. |

    Methods in org.cloudbus.cloudsim that return types with arguments of type Vm

    | Modifier and Type | Method and Description |
    | --- | --- |
    |  |  |
    | --- | --- |
    | `java.util.List<Vm>` | HostDynamicWorkload.`getCompletedVms()` Gets the completed vms. |
    | `java.util.List<Vm>` | Host.`getVmsMigratingIn()` Gets the vms migrating in. |

    Methods in org.cloudbus.cloudsim with parameters of type Vm

    | Modifier and Type | Method and Description |
    | --- | --- |
    |  |  |
    | --- | --- |
    | `void` | Host.`addMigratingInVm(Vm vm)` Adds the migrating in vm. |
    | `abstract boolean` | VmAllocationPolicy.`allocateHostForVm(Vm vm)` Allocates a host for a given VM. |
    | `boolean` | VmAllocationPolicySimple.`allocateHostForVm(Vm vm)` Allocates a host for a given VM. |
    | `abstract boolean` | VmAllocationPolicy.`allocateHostForVm(Vm vm, Host host)` Allocates a specified host for a given VM. |
    | `boolean` | VmAllocationPolicySimple.`allocateHostForVm(Vm vm, Host host)` |
    | `boolean` | Host.`allocatePesForVm(Vm vm, java.util.List<java.lang.Double> mipsShare)` Allocates PEs for a VM. |
    | `abstract boolean` | VmScheduler.`allocatePesForVm(Vm vm, java.util.List<java.lang.Double> mipsShare)` Allocates PEs for a VM. |
    | `boolean` | VmSchedulerSpaceShared.`allocatePesForVm(Vm vm, java.util.List<java.lang.Double> mipsShare)` |
    | `boolean` | VmSchedulerTimeShared.`allocatePesForVm(Vm vm, java.util.List<java.lang.Double> mipsShareRequested)` |
    | `abstract void` | VmAllocationPolicy.`deallocateHostForVm(Vm vm)` Releases the host used by a VM. |
    | `void` | VmAllocationPolicySimple.`deallocateHostForVm(Vm vm)` Releases the host used by a VM. |
    | `void` | Host.`deallocatePesForVm(Vm vm)` Releases PEs allocated to a VM. |
    | `abstract void` | VmScheduler.`deallocatePesForVm(Vm vm)` Releases PEs allocated to a VM. |
    | `void` | VmSchedulerSpaceShared.`deallocatePesForVm(Vm vm)` |
    | `void` | VmSchedulerTimeShared.`deallocatePesForVm(Vm vm)` |
    | `java.util.List<java.lang.Double>` | Host.`getAllocatedMipsForVm(Vm vm)` Returns the MIPS share of each Pe that is allocated to a given VM. |
    | `java.util.List<java.lang.Double>` | VmScheduler.`getAllocatedMipsForVm(Vm vm)` Returns the MIPS share of each Pe that is allocated to a given VM. |
    | `abstract Host` | VmAllocationPolicy.`getHost(Vm vm)` Get the host that is executing the given VM belonging to the given user. |
    | `Host` | VmAllocationPolicySimple.`getHost(Vm vm)` Gets the host that is executing the given VM belonging to the given user. |
    | `double` | HostDynamicWorkload.`getMaxUtilizationAmongVmsPes(Vm vm)` Gets the max utilization among by all PEs allocated to the VM. |
    | `java.util.List<Pe>` | VmScheduler.`getPesAllocatedForVM(Vm vm)` Gets the pes allocated for vm. |
    | `double` | Host.`getTotalAllocatedMipsForVm(Vm vm)` Gets the total allocated MIPS for a VM over all the PEs. |
    | `double` | VmScheduler.`getTotalAllocatedMipsForVm(Vm vm)` Gets the total allocated MIPS for a VM over all the PEs. |
    | `boolean` | Host.`isSuitableForVm(Vm vm)` Checks if is suitable for vm. |
    | `void` | Host.`removeMigratingInVm(Vm vm)` Removes the migrating in vm. |
    | `boolean` | Host.`vmCreate(Vm vm)` Allocates PEs and memory to a new VM in the Host. |
    | `protected void` | Host.`vmDeallocate(Vm vm)` Deallocate all hostList for the VM. |
    | `void` | Host.`vmDestroy(Vm vm)` Destroys a VM running in the host. |

    Method parameters in org.cloudbus.cloudsim with type arguments of type Vm

    | Modifier and Type | Method and Description |
    | --- | --- |
    |  |  |
    | --- | --- |
    | `abstract java.util.List<java.util.Map<java.lang.String,java.lang.Object>>` | VmAllocationPolicy.`optimizeAllocation(java.util.List<? extends Vm> vmList)` Optimize allocation of the VMs according to current utilization. |
    | `java.util.List<java.util.Map<java.lang.String,java.lang.Object>>` | VmAllocationPolicySimple.`optimizeAllocation(java.util.List<? extends Vm> vmList)` |
    | `void` | DatacenterBroker.`submitVmList(java.util.List<? extends Vm> list)` This method is used to send to the broker the list with virtual machines that must be created. |
  - ### Uses of Vm in org.cloudbus.cloudsim.examples

    Methods in org.cloudbus.cloudsim.examples that return types with arguments of type Vm

    | Modifier and Type | Method and Description |
    | --- | --- |
    |  |  |
    | --- | --- |
    | `java.util.List<Vm>` | CloudSimExample8.GlobalBroker.`getVmList()` |

    Method parameters in org.cloudbus.cloudsim.examples with type arguments of type Vm

    | Modifier and Type | Method and Description |
    | --- | --- |
    |  |  |
    | --- | --- |
    | `protected void` | CloudSimExample8.GlobalBroker.`setVmList(java.util.List<Vm> vmList)` |
  - ### Uses of Vm in org.cloudbus.cloudsim.examples.power

    Fields in org.cloudbus.cloudsim.examples.power with type parameters of type Vm

    | Modifier and Type | Field and Description |
    | --- | --- |
    |  |  |
    | --- | --- |
    | `protected static java.util.List<Vm>` | RunnerAbstract.`vmList` The vm list. |

    Methods in org.cloudbus.cloudsim.examples.power that return types with arguments of type Vm

    | Modifier and Type | Method and Description |
    | --- | --- |
    |  |  |
    | --- | --- |
    | `static java.util.List<Vm>` | Helper.`createVmList(int brokerId, int vmsNumber)` Creates the vm list. |

    Method parameters in org.cloudbus.cloudsim.examples.power with type arguments of type Vm

    | Modifier and Type | Method and Description |
    | --- | --- |
    |  |  |
    | --- | --- |
    | `protected static java.util.Map<java.lang.String,java.lang.Double>` | Helper.`getSlaMetrics(java.util.List<Vm> vms)` Gets the sla metrics. |
    | `static java.util.List<java.lang.Double>` | Helper.`getTimesBeforeVmMigration(java.util.List<Vm> vms)` Gets the times before vm migration. |
    | `static void` | Helper.`printResults(PowerDatacenter datacenter, java.util.List<Vm> vms, double lastClock, java.lang.String experimentName, boolean outputInCsv, java.lang.String outputFolder)` Prints the results. |
  - ### Uses of Vm in org.cloudbus.cloudsim.lists

    Methods in org.cloudbus.cloudsim.lists with type parameters of type Vm

    | Modifier and Type | Method and Description |
    | --- | --- |
    |  |  |
    | --- | --- |
    | `static <T extends Vm>  T` | VmList.`getById(java.util.List<T> vmList, int id)` Return a reference to a Vm object from its ID. |
    | `static <T extends Vm>  T` | VmList.`getByIdAndUserId(java.util.List<T> vmList, int id, int userId)` Return a reference to a Vm object from its ID and user ID. |

    Methods in org.cloudbus.cloudsim.lists with parameters of type Vm

    | Modifier and Type | Method and Description |
    | --- | --- |
    |  |  |
    | --- | --- |
    | `static <T extends Pe>  double` | PeList.`getMaxUtilizationAmongVmsPes(java.util.List<T> peList, Vm vm)` Gets the max utilization among by all PEs allocated to the VM. |
  - ### Uses of Vm in org.cloudbus.cloudsim.network.datacenter

    Subclasses of Vm in org.cloudbus.cloudsim.network.datacenter

    | Modifier and Type | Class and Description |
    | --- | --- |
    |  |  |
    | --- | --- |
    | `class` | `NetworkVm` NetworkVm class extends Vm to support simulation of networked datacenters. |

    Fields in org.cloudbus.cloudsim.network.datacenter with type parameters of type Vm

    | Modifier and Type | Field and Description |
    | --- | --- |
    |  |  |
    | --- | --- |
    | `java.util.List<Vm>` | Switch.`BagofTaskVm` |

    Methods in org.cloudbus.cloudsim.network.datacenter with type parameters of type Vm

    | Modifier and Type | Method and Description |
    | --- | --- |
    |  |  |
    | --- | --- |
    | `<T extends Vm>  java.util.List<T>` | NetDatacenterBroker.`getVmList()` Gets the vm list. |
    | `<T extends Vm>  java.util.List<T>` | NetDatacenterBroker.`getVmsCreatedList()` Gets the vm list. |
    | `protected <T extends Vm>  void` | NetDatacenterBroker.`setVmList(java.util.List<T> vmList)` Sets the vm list. |
    | `protected <T extends Vm>  void` | NetDatacenterBroker.`setVmsCreatedList(java.util.List<T> vmsCreatedList)` Sets the vm list. |

    Methods in org.cloudbus.cloudsim.network.datacenter with parameters of type Vm

    | Modifier and Type | Method and Description |
    | --- | --- |
    |  |  |
    | --- | --- |
    | `boolean` | NetworkVmAllocationPolicy.`allocateHostForVm(Vm vm)` Allocates a host for a given VM. |
    | `boolean` | NetworkVmAllocationPolicy.`allocateHostForVm(Vm vm, Host host)` |
    | `void` | NetworkVmAllocationPolicy.`deallocateHostForVm(Vm vm)` Releases the host used by a VM. |
    | `Host` | NetworkVmAllocationPolicy.`getHost(Vm vm)` Gets the host that is executing the given VM belonging to the given user. |
    | `protected double` | NetworkVmAllocationPolicy.`getMaxUtilizationAfterAllocation(NetworkHost host, Vm vm)` |
    | `double` | NetworkHost.`getMaxUtilizationAmongVmsPes(Vm vm)` |
    | `boolean` | NetworkDatacenter.`processVmCreateNetwork(Vm vm)` Create the VM within the NetworkDatacenter. |

    Method parameters in org.cloudbus.cloudsim.network.datacenter with type arguments of type Vm

    | Modifier and Type | Method and Description |
    | --- | --- |
    |  |  |
    | --- | --- |
    | `java.util.List<java.util.Map<java.lang.String,java.lang.Object>>` | NetworkVmAllocationPolicy.`optimizeAllocation(java.util.List<? extends Vm> vmList)` |
    | `void` | NetDatacenterBroker.`submitVmList(java.util.List<? extends Vm> list)` This method is used to send to the broker the list with virtual machines that must be created. |
  - ### Uses of Vm in org.cloudbus.cloudsim.power

    Subclasses of Vm in org.cloudbus.cloudsim.power

    | Modifier and Type | Class and Description |
    | --- | --- |
    |  |  |
    | --- | --- |
    | `class` | `PowerVm` The class of a VM that stores its CPU utilization history. |

    Methods in org.cloudbus.cloudsim.power that return Vm

    | Modifier and Type | Method and Description |
    | --- | --- |
    |  |  |
    | --- | --- |
    | `abstract Vm` | PowerVmSelectionPolicy.`getVmToMigrate(PowerHost host)` Gets the vms to migrate. |
    | `Vm` | PowerVmSelectionPolicyMaximumCorrelation.`getVmToMigrate(PowerHost host)` |
    | `Vm` | PowerVmSelectionPolicyMinimumMigrationTime.`getVmToMigrate(PowerHost host)` |
    | `Vm` | PowerVmSelectionPolicyMinimumUtilization.`getVmToMigrate(PowerHost host)` |
    | `Vm` | PowerVmSelectionPolicyRandomSelection.`getVmToMigrate(PowerHost host)` |

    Methods in org.cloudbus.cloudsim.power that return types with arguments of type Vm

    | Modifier and Type | Method and Description |
    | --- | --- |
    |  |  |
    | --- | --- |
    | `protected java.util.List<? extends Vm>` | PowerVmAllocationPolicyMigrationAbstract.`getVmsToMigrateFromHosts(java.util.List<PowerHostUtilizationHistory> overUtilizedHosts)` Gets the vms to migrate from hosts. |
    | `protected java.util.List<? extends Vm>` | PowerVmAllocationPolicyMigrationAbstract.`getVmsToMigrateFromUnderUtilizedHost(PowerHost host)` Gets the vms to migrate from under utilized host. |

    Methods in org.cloudbus.cloudsim.power with parameters of type Vm

    | Modifier and Type | Method and Description |
    | --- | --- |
    |  |  |
    | --- | --- |
    | `boolean` | PowerVmAllocationPolicyAbstract.`allocateHostForVm(Vm vm)` |
    | `boolean` | PowerVmAllocationPolicyAbstract.`allocateHostForVm(Vm vm, Host host)` |
    | `void` | PowerVmAllocationPolicyAbstract.`deallocateHostForVm(Vm vm)` |
    | `PowerHost` | PowerVmAllocationPolicyAbstract.`findHostForVm(Vm vm)` Find host for vm. |
    | `PowerHost` | PowerVmAllocationPolicyMigrationAbstract.`findHostForVm(Vm vm)` Find host for vm. |
    | `PowerHost` | PowerVmAllocationPolicyMigrationAbstract.`findHostForVm(Vm vm, java.util.Set<? extends Host> excludedHosts)` Find host for vm. |
    | `Host` | PowerVmAllocationPolicyAbstract.`getHost(Vm vm)` |
    | `protected double` | PowerVmAllocationPolicyMigrationAbstract.`getMaxUtilizationAfterAllocation(PowerHost host, Vm vm)` Gets the power after allocation. |
    | `protected double` | PowerVmAllocationPolicyMigrationAbstract.`getPowerAfterAllocation(PowerHost host, Vm vm)` Gets the power after allocation. |
    | `protected boolean` | PowerVmAllocationPolicyMigrationAbstract.`isHostOverUtilizedAfterAllocation(PowerHost host, Vm vm)` Checks if is host over utilized after allocation. |

    Method parameters in org.cloudbus.cloudsim.power with type arguments of type Vm

    | Modifier and Type | Method and Description |
    | --- | --- |
    |  |  |
    | --- | --- |
    | `protected java.util.List<java.util.Map<java.lang.String,java.lang.Object>>` | PowerVmAllocationPolicyMigrationAbstract.`getNewVmPlacement(java.util.List<? extends Vm> vmsToMigrate, java.util.Set<? extends Host> excludedHosts)` Gets the new vm placement. |
    | `protected java.util.List<java.util.Map<java.lang.String,java.lang.Object>>` | PowerVmAllocationPolicyMigrationAbstract.`getNewVmPlacementFromUnderUtilizedHost(java.util.List<? extends Vm> vmsToMigrate, java.util.Set<? extends Host> excludedHosts)` Gets the new vm placement from under utilized host. |
    | `java.util.List<java.util.Map<java.lang.String,java.lang.Object>>` | PowerVmAllocationPolicyMigrationAbstract.`optimizeAllocation(java.util.List<? extends Vm> vmList)` Optimize allocation of the VMs according to current utilization. |
    | `java.util.List<java.util.Map<java.lang.String,java.lang.Object>>` | PowerVmAllocationPolicySimple.`optimizeAllocation(java.util.List<? extends Vm> vmList)` |
  - ### Uses of Vm in org.cloudbus.cloudsim.power.lists

    Methods in org.cloudbus.cloudsim.power.lists with type parameters of type Vm

    | Modifier and Type | Method and Description |
    | --- | --- |
    |  |  |
    | --- | --- |
    | `static <T extends Vm>  void` | PowerVmList.`sortByCpuUtilization(java.util.List<T> vmList)` Sort by cpu utilization. |
  - ### Uses of Vm in org.cloudbus.cloudsim.provisioners

    Methods in org.cloudbus.cloudsim.provisioners with parameters of type Vm

    | Modifier and Type | Method and Description |
    | --- | --- |
    |  |  |
    | --- | --- |
    | `abstract boolean` | BwProvisioner.`allocateBwForVm(Vm vm, long bw)` Allocates BW for a given VM. |
    | `boolean` | BwProvisionerSimple.`allocateBwForVm(Vm vm, long bw)` |
    | `abstract boolean` | PeProvisioner.`allocateMipsForVm(Vm vm, double mips)` Allocates MIPS for a given VM. |
    | `boolean` | PeProvisionerSimple.`allocateMipsForVm(Vm vm, double mips)` |
    | `abstract boolean` | PeProvisioner.`allocateMipsForVm(Vm vm, java.util.List<java.lang.Double> mips)` Allocates MIPS for a given VM. |
    | `boolean` | PeProvisionerSimple.`allocateMipsForVm(Vm vm, java.util.List<java.lang.Double> mips)` |
    | `abstract boolean` | RamProvisioner.`allocateRamForVm(Vm vm, int ram)` Allocates RAM for a given VM. |
    | `boolean` | RamProvisionerSimple.`allocateRamForVm(Vm vm, int ram)` |
    | `abstract void` | BwProvisioner.`deallocateBwForVm(Vm vm)` Releases BW used by a VM. |
    | `void` | BwProvisionerSimple.`deallocateBwForVm(Vm vm)` |
    | `abstract void` | PeProvisioner.`deallocateMipsForVm(Vm vm)` Releases MIPS used by a VM. |
    | `void` | PeProvisionerSimple.`deallocateMipsForVm(Vm vm)` |
    | `abstract void` | RamProvisioner.`deallocateRamForVm(Vm vm)` Releases BW used by a VM. |
    | `void` | RamProvisionerSimple.`deallocateRamForVm(Vm vm)` |
    | `abstract long` | BwProvisioner.`getAllocatedBwForVm(Vm vm)` Gets the allocated BW for VM. |
    | `long` | BwProvisionerSimple.`getAllocatedBwForVm(Vm vm)` |
    | `abstract java.util.List<java.lang.Double>` | PeProvisioner.`getAllocatedMipsForVm(Vm vm)` Gets allocated MIPS for a given VM. |
    | `java.util.List<java.lang.Double>` | PeProvisionerSimple.`getAllocatedMipsForVm(Vm vm)` |
    | `abstract double` | PeProvisioner.`getAllocatedMipsForVmByVirtualPeId(Vm vm, int peId)` Gets allocated MIPS for a given VM for a given virtual Pe. |
    | `double` | PeProvisionerSimple.`getAllocatedMipsForVmByVirtualPeId(Vm vm, int peId)` |
    | `abstract int` | RamProvisioner.`getAllocatedRamForVm(Vm vm)` Gets the allocated RAM for VM. |
    | `int` | RamProvisionerSimple.`getAllocatedRamForVm(Vm vm)` |
    | `abstract double` | PeProvisioner.`getTotalAllocatedMipsForVm(Vm vm)` Gets total allocated MIPS for a given VM for all PEs. |
    | `double` | PeProvisionerSimple.`getTotalAllocatedMipsForVm(Vm vm)` |
    | `abstract boolean` | RamProvisioner.`isSuitableForVm(Vm vm, int ram)` Checks if is suitable for vm. |
    | `boolean` | RamProvisionerSimple.`isSuitableForVm(Vm vm, int ram)` |
    | `abstract boolean` | BwProvisioner.`isSuitableForVm(Vm vm, long bw)` Checks if BW is suitable for vm. |
    | `boolean` | BwProvisionerSimple.`isSuitableForVm(Vm vm, long bw)` |
  - ### Uses of Vm in org.workflowsim

    Subclasses of Vm in org.workflowsim

    | Modifier and Type | Class and Description |
    | --- | --- |
    |  |  |
    | --- | --- |
    | `class` | `CondorVM` Condor Vm extends a VM: the difference is it has a locl storage system and it has a state to indicate whether it is busy or not |

    Fields in org.workflowsim with type parameters of type Vm

    | Modifier and Type | Field and Description |
    | --- | --- |
    |  |  |
    | --- | --- |
    | `protected java.util.List<? extends Vm>` | WorkflowEngine.`vmList` |

    Methods in org.workflowsim with type parameters of type Vm

    | Modifier and Type | Method and Description |
    | --- | --- |
    |  |  |
    | --- | --- |
    | `<T extends Vm>  java.util.List<T>` | WorkflowEngine.`getVmList()` Gets the vm list. |

    Methods in org.workflowsim that return types with arguments of type Vm

    | Modifier and Type | Method and Description |
    | --- | --- |
    |  |  |
    | --- | --- |
    | `java.util.List<? extends Vm>` | WorkflowEngine.`getAllVmList()` |

    Method parameters in org.workflowsim with type arguments of type Vm

    | Modifier and Type | Method and Description |
    | --- | --- |
    |  |  |
    | --- | --- |
    | `void` | WorkflowEngine.`submitVmList(java.util.List<? extends Vm> list)` |
    | `void` | WorkflowEngine.`submitVmList(java.util.List<? extends Vm> list, int schedulerId)` This method is used to send to the broker the list with virtual machines that must be created. |

- Overview
- Package
- Class
- Use
- Tree
- Deprecated
- Index
- Help

- Prev
- Next

- Frames
- No Frames

- All Classes
